# Nonlinear Adaptive PID Control for Greenhouse Environment Based on RBF Network

**DOI:** 10.3390/s120505328

**Published:** 2012-04-26

**Authors:** Songwei Zeng, Haigen Hu, Lihong Xu, Guanghui Li

**Affiliations:** 1 School of Information Engineering, Zhejiang Agriculture & Forestry University, Lin'an 311300, China; E-Mails: zsw@zafu.edu.cn (S.Z.), lgh@zafu.edu.cn (G.L.); 2 Department of Control Science and Engineering, Tongji University, Shanghai 200092, China

**Keywords:** nonlinear adaptive control, neuro-PID control, Radial Basis Function (RBF), greenhouse environment control, Genetic Algorithm (GA)

## Abstract

This paper presents a hybrid control strategy, combining Radial Basis Function (RBF) network with conventional proportional, integral, and derivative (PID) controllers, for the greenhouse climate control. A model of nonlinear conservation laws of enthalpy and matter between numerous system variables affecting the greenhouse climate is formulated. RBF network is used to tune and identify all PID gain parameters online and adaptively. The presented Neuro-PID control scheme is validated through simulations of set-point tracking and disturbance rejection. We compare the proposed adaptive online tuning method with the offline tuning scheme that employs Genetic Algorithm (GA) to search the optimal gain parameters. The results show that the proposed strategy has good adaptability, strong robustness and real-time performance while achieving satisfactory control performance for the complex and nonlinear greenhouse climate control system, and it may provide a valuable reference to formulate environmental control strategies for actual application in greenhouse production.

## 1. Introduction

The greenhouse climate control concerns the creation of a favorable environment for the crop in order to reach predetermined results for high yield, high quality and low production costs. However, it is a very difficult to implement in practice due to the complexity of the greenhouse environments. Greenhouses are highly nonlinear and strongly coupled Multi-Input Multi-Output (MIMO) systems that are largely influenced by the outside weather (wind velocity, outside temperature and humidity) and by many other practical constraints (actuators and moistening cycle). In recent years, various advanced control techniques and related strategies, such as predictive control [1–3], adaptive control [4,5], nonlinear feedback control [6], fuzzy control [7,8], robust control [9], optimal control [10–12] and compatible control [13] have been proposed for greenhouse environment control. These studies are very important to real-world engineering application in greenhouse production. However, most of these approaches are either theoretically complex or difficult to implement in actual greenhouse production. Controller designs for greenhouse environmental control mostly adopt conventional proportional, integral, and derivative (PID) controllers owing to the simple architecture, easy implementation and excellent performance.

About 95% of the regulatory controllers of the process control [14], motor drives, automotive, fight control and instrumentation industries have PID structures. In spite of this widespread usage, their effectiveness is often limited owing to poor tuning, and tuning PID controllers efficiently is the subject of active research. Several tuning methods have been presented in the existing literature [15,16]; these include designs based on “guess-and-check” such as trial and error, based on linear control theory such as Ziegler–Nichols (Z-N) and Cohen–Coon methods (C-C). Nevertheless, it is difficult to achieve desired performance of the controlled greenhouse using conventional tuning methods because there is nearly no effective analytical way to find the optimal set of gain parameters. The methods are mostly based on linear models, which are usually adjusted around operating points. Empirical methods such as Z-N, which can be used for tuning a simple problem, are inadequate to deal with complex systems like the greenhouse environment owing to the lack of empirical data for a wide range of problems. Hence, new designs for tuning PID parameters should be explored to regulate the greenhouse environment. A conventional fixed gain or gain-scheduled PID controller may assure stability for this maneuver, but it is difficult to achieve the desired performance for controlling nonlinear and strong interference systems due to the difficulties in determining appropriate PID gains.

Recently, an optimal tuning method of PID controller employing Genetic Algorithm (GA) has been proposed and successfully used for a wide range of plants [17–19]. Due to the capability for global and powerful optimization, GA can search optimal PID gain parameters based on various performance criterion for many different control problems. However, a limitation exists in the computational cost of the optimization process and in the heavy dependence on the computation time when searching the optimal gain parameters. Therefore this method cannot be applied to online real-time control. Besides, it is important that the aforementioned methods cannot adapt PID gain parameters properly to prevailing dynamics owing to the presence of varying strong disturbances. Hence, new designs for self-adaptive online tuning of PID parameters should be explored to control these systems.

Design methods incorporating artificial neural networks (ANNs) have been widely applied in the area of nonlinear adaptive control due to their adaptability, learning ability and powerful ability to approximate nonlinear functions [20–31]. Among the various neural network paradigms, the Radial Basis Function (RBF) network has been extensively studied in the area of modeling, prediction and control for nonlinear systems due to its attractive properties such as localization, functional approximation, cluster modeling, quasi-orthogonality and a simple network structure. In [23], an on-line learning neuro-control scheme that incorporates a growing RBF network (GRBFN) is proposed for a non-linear aircraft controller design. In [24], an adaptive control scheme was studied for a 1-DOF motion model based on the RBF approximations. In [27], RBF network was used to model the inside air temperature as a function of the outside air temperature and solar radiation, and inside relative humidity in a hydroponic greenhouse. In [31], a Functional electrical stimulation (FES) system tuned by Radial Basis Function (RBF) Neural Network-based Proportional-Integral-Derivative (PID) model was designed to control the knee joint according to the desired trajectory through stimulation of lower limbs muscles.

Motivated by the aforementioned issues, an adaptive neural control scheme, incorporating the conventional PID controllers, is presented herein for greenhouse climate control. It is anticipated that the combination will take advantage of the simplicity of PID controllers and the powerful capability of learning and adaptability of RBF networks. The main objective is to develop an online adaptive tuning method by employing RBF networks for greenhouse climate control with two PID loops of a MIMO process, which is characterized by strong interactions among process variables, nonlinearities and serious interference. In order to validate the effectiveness and superiority of the proposed adaptive control scheme, we compare the results with an offline tuning method that adopts Genetic Algorithm (GA) to search the conventional PID gain parameters based on criteria of integral time absolute error (ITAE).

The remainder of this paper is organized as follows. Section 2 describes the considered greenhouse climate dynamic model and the corresponding nonlinear differential equations. Section 3 describes RBF network structure, the corresponding control strategy, and the adaptive tuning scheme based on Jacobian identification of RBF network. Section 4 presents simulations and results, and the proposed adaptive control scheme is compared with an offline tuning method that uses GA to search the optimal gain parameters. Finally, conclusions and prospects for this work are given in Section 5.

## 2. Description and Problem Formulation

### 2.1. Greenhouse Dynamic Model

The greenhouse environment is a complex dynamic system. Over the past decades, people have gained a considerable understanding of greenhouse climate dynamics, and many methods describing the dynamic process of a greenhouse climate have been proposed. Traditionally, there are two different approaches to describe the greenhouse climate: one is based on energy and mass flow equations describing the process [32–35], and another is based on the analysis of input-output data from the process by using a system identification approach [27,36–38]. This paper deals with the first method for the control of inside air temperature and humidity of a greenhouse, and its physical model describes flow and mass transfers generated by the differences in energy and mass content between the inside and outside air, by control, or by exogenous energy and mass inputs [39]. Most analytic models on analysis and control of the greenhouses environment have been based on the following state space form:

(1) $$\dot{x}=f(t,x,u,\upsilon )$$

where *x* are states variables such as indoor temperature, humidity and carbon dioxide concentration; *u* are control inputs such as energy input by the heating system, fogging systems, ventilation system and *CO_2_* supply flux; *v* are external disturbances such as solar radiation, outdoor temperature, humidity and wind speed; *t* denotes time, and *f*(*·*) is a nonlinear function.

**Figure 1.**
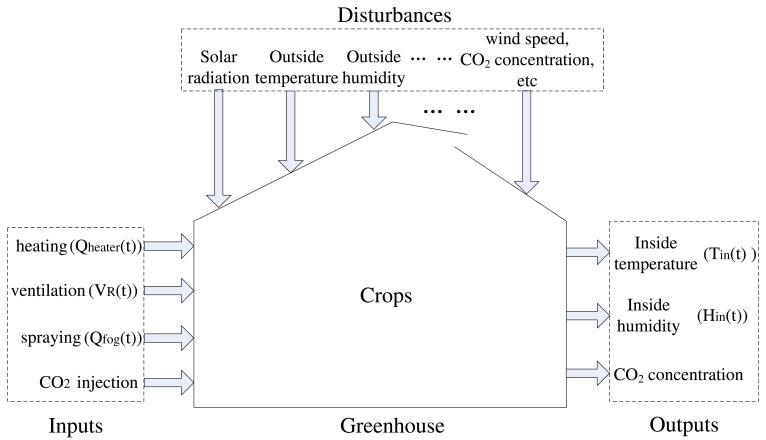
Greenhouse climate dynamic model.

In order to effectively validate the performance of the proposed algorithm, the considered greenhouse analytic expression is based on the heating/cooling/ventilating model, which can be obtained from several references [6,40-42]. This can be summarized in the functional block diagram given in Figure 1. Considering the related high costs, *CO_2_* supply systems are not extensively used, therefore the related variables are not taken into account in this work. To simplify the model, we consider only primary disturbance variables, such as solar radiation, outside temperature and humidity. According to the above analysis, the state equations have been formed based on the laws of conservation of enthalpy and matter, and the dynamic behaviour of states is described by using the following differential equations [42]:

(2) $$\frac{dT_{in}(t)}{dt}=\frac{1}{\rho C_{p}V_{T}}(Q_{\text{heater}}(t)+S_{i}(t)-\lambda Q_{\text{fog}}(t))-\left(\frac{V_{R}(t)}{V_{T}}+\frac{UA}{\rho C_{p}V_{T}}\right)(T_{in}(t)-T_{\text{out}}(t))$$

(3) $$\frac{dH_{in}(t)}{dt}=\frac{1}{\rho V_{H}}Q_{\text{fog}}(t)+\frac{1}{\rho V_{H}}(E(S_{i}(t),H_{in}(t)))-\frac{V_{R}(t)}{\rho V_{H}}(H_{in}(t)-H_{\text{out}}(t)$$

where

*T_in_/T_out_* is the indoor/outdoor air temperature (°C),
*H_in_/H_out_* is the interior/exterior humidity ratio (*g*[*H_2_O*]*kg-^1^*[*dry* air]),
*UA* is the heat transfer coefficient of enclosure (*WK^-^*^1^),
*V* is the geometric volume of the greenhouse (*m*^3^),
*ρ* is the air density (*kg*[air]*m*-^3^),
*C_p_* is the specific heat of air (*Jkg^−^*^1^*K^−1^*),
*Q_heater_* is the heat provided by the greenhouse heater (*W*),
*Q_fog_* is the water capacity of the fog system (*g*[*H_2_O*]*s^−1^*)
*S_i_* is the intercepted solar radiant energy (*W*),
*λ* is the latent heat of vaporization (*Jg^−^*^1^),
*V_R_* is the ventilation rate (*m*^3^[air]*s^−^*^1^),
*E*(*S_i_*(*t*), *H_in_*(*t*)) is the evapotranspiration rate of the plants (*g*[*H_2_O*]*s^−1^*),

*V_T_* and *V_H_* are the active mixing air volumes of the temperature and humidity respectively Generally speaking, *V_T_* and *V_H_* are as small as 60%-70% of the geometric volume *V* of the greenhouse owing to local convection and stagnant zones that exist in ventilated spaces.

It is also worth noting that as a first approximation, the evapotranspiration rate *E*(*S_i_, H_in_*) is primarily related to the intercepted solar radiant energy, through the following simplified relation:

(4) $$E(S_{i}(t),H_{in}(t))=\alpha \frac{S_{i}(t)}{\lambda }-\beta_{T}H_{in}(t)$$

where *α* is an overall coefficient to account for shading and leaf area index (dimensionless) and *β_T_* is the overall coefficient to account for thermodynamic constants and other factors affecting evapotranspiration (*i.e.*,stomata and air motion) [42].

### 2.2. Problem Formulation

The climate model provided above can be used in all seasons, and two variables are controlled, namely, the indoor air temperature and the humidity ratio through the processes of heating (*Q_heater_*(*t*)), ventilation (*V_R_*(*t*)) and fogging (*Q_fog_*(*t*)). For summer operation in this work, *Q_heater_* is set to zero. The purposes of ventilation are to exhaust moist air and to replace it with outside fresh air, to control high temperatures caused by the influx of solar radiation, to dehumidify the greenhouse air when the humidity of the outside air is very low, to provide uniform air flow throughout the entire greenhouse, and to maintain acceptable levels of gas concentration in the greenhouse. Fogging systems (such as misters, fog units, or roof sprinklers) are primarily used for humidification of the greenhouse. In fact, fogging system also plays a cooling role due to evaporative cooling. Moreover, fresh air must be continually ventilated into the greenhouse, while warm and humidified air is exhausted. When humidification occurs under sunny conditions, ventilation is necessary since the greenhouse would soon become a steam bath if fresh dry air is not provided.

In order to effectively express the state-space form, we define the inside temperature and absolute humidity as the dynamic state variables, *x*_1_(*t*) and *x_2_*(*t*), respectively, the ventilation rate and the water capacity of the fog system as the control (actuator) variables, *u*_1_(*t*) and *u_2_*(*t*), respectively, and the intercepted solar radiant energy, the outside temperature, and the outside absolute humidity as the disturbances, *v_i_*(*t*), *i =* 1,2,3. Equations (2) and (3) can alternatively be written in the following state-space form:

(5) $${\dot{x}}_{1}(t)=\frac{UA}{\rho C_{p}V_{T}}x_{1}(t)-\frac{1}{V_{T}}x_{1}(t)u_{1}(t)-\frac{\lambda }{\rho C_{p}V_{T}}u_{2}(t)+\frac{1}{\rho C_{P}V_{T}}\upsilon_{1}(t)+\frac{UA}{\rho C_{p}V_{T}}u_{1}(t)\upsilon_{2}(t)+\frac{1}{V_{T}}u_{1}(t)\upsilon_{2}(t)$$

(6) $${\dot{x}}_{2}(t)=\frac{\beta_{T}}{\rho V_{H}}x_{2}(t)+\frac{1}{\rho V_{H}}u_{2}(t)+\frac{\alpha }{\lambda \rho V_{H}}\upsilon_{1}(t)+\frac{1}{\rho V_{H}}x_{2}(t)u_{1}(t)+\frac{1}{\rho V_{H}}u_{1}(t)\upsilon_{3}(t)$$

Due to the complexity appearing as the cross-product terms between control and disturbance variables, Equations (5) and (6) are obviously coupled nonlinear equations, which cannot be placed into the more familiar form of an affine analytic nonlinear system.

## 3. Design of Adaptive Neuro-PID Controller for Greenhouse Climate

### 3.1. RBF Network Structure

**Figure 2.**
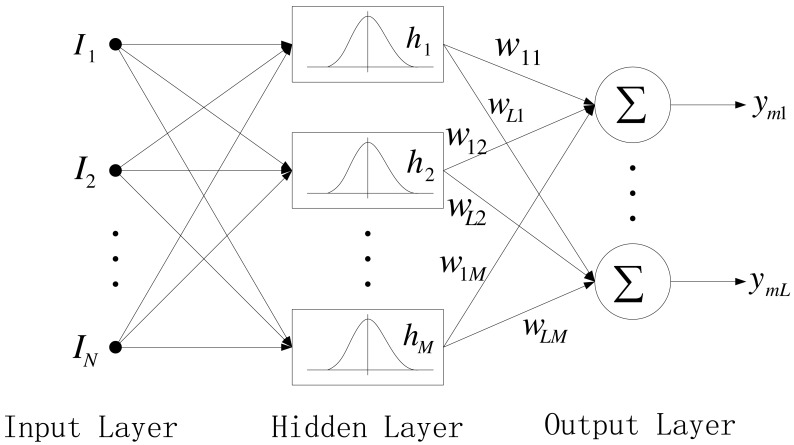
Architecture of a RBF network.

RBF, presented by J. Moody and C. Darken [43], emerged as a variant of artificial neural network in late 1980s. RBF neural networks have an input layer, a hidden layer and an output layer. The neurons in the hidden layer contain Gaussian transfer functions whose outputs are inversely proportional to the distance from the center of the neuron. The architecture of a typical RBF network is shown in Figure 2.

Each input node corresponds to an element of the input vector *I* ∊ *R^n^*, and each hidden node implements a radial activated function, which consists of local perception nodes. The Gaussian activation function for RBF network is given by:

(7) $$h_{j}=\exp\left(\frac{\left\|I-C_{j}\right\|}{2\sigma_{j}^{2}}\right)\;j=1,2,\ldots ,M$$

where *I* = [*I*_1_, *I_2_,…, I_N_*] and *M* are the input feature vector and the number of hidden nodes, respectively. *C_j_ =* [*c_j_*_1_,*…, c_jN_*] is *N*-dimensional center parameter of the *j*th hidden node, symbol ‖ • ‖ denotes the Euclidean norm, and *σ_j_* is the positive center width parameter.

The output nodes implement a weighted sum of hidden node outputs as follows:

(8) $$y_{mi}=\sum_{j=1}^{M}w_{ij}h_{j}\;i=1,2,\ldots ,L$$

where *w_ij_* is the connection weight of the *j*th hidden node to the *i*th output node, and *L* is the number of output nodes.

### 3.2. Control Strategy

In order to simulate its behavior on a digital computer, we adopt a fourth-order Runge-Kutta numerical method with a small enough integration step. Hence, considering a typical digital positional PID algorithm is generally given as:

(9) $$u(k)=u_{0}+K_{p}e(k)+K_{i}T_{s}\sum_{i=1}^{k}e(i)+K_{d}\frac{e(k)-e(k-1)}{T_{s}}$$

where *k* and *T_s_* are iterative step and sampling time, respectively. *u*(*k*) and *u*_0_ are the control law and the corresponding initial value. *K_p_, K_i_* and *K_d_* are the gains of the proportional, integral and derivative terms of a PID controller, respectively. *e*(*k*) is the process tracking error, defined as:

(10) e(k)=r(k)−y(k)

with *r*(*k*) and *y*(*k*) being the desired trajectory and the process output, respectively.

**Figure 3.**
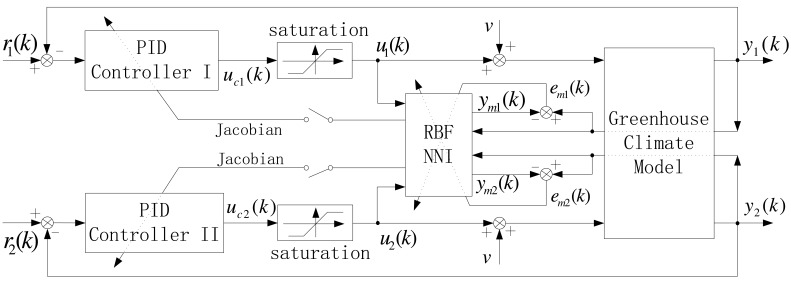
Block diagram of Neuro-PID greenhouse climate control system.

Note that the greenhouse dynamic system mentioned above is a two-input and two-output continuous time nonlinear system. We adopt a hybrid control strategy by combining RBF network with the conventional PID controller, in which there is one neural network model with two outputs (namely, inside temperature and humidity). The corresponding control structure is shown in Figure 3, which is similar to the references [28-31]. RBF network is used to tune the parameters of the conventional PID controller through Jacobian information. To facilitate the subsequent development, the following parameter vectors are only for single loop operation if there does not exist an explicit declaration in the text, and the vectors related to the controller parameter are defined as:

(11) $$K_{c}(k)=\left(K_{p}(k)K_{i}(k)K_{d}(k)\right)$$

(12) $$e_{c}(k)={\left(e(k)T_{s}\sum_{i=1}^{k}e(i)\frac{e(k)-e(k-1)}{T_{s}}\right)}^{T}$$

Considering the saturation of actuators, the control law Equation (9) is rewritten as:

(13) $$u(k)=\{\begin{array}{ll} u_{\text{lim}} & \text{if}\left\|u_{c}\right\|\geq u_{\text{lim}}\\ u_{0}+K_{c}(k)e_{c}(k), & \text{otherwise} \end{array}$$

where *u_lim_* is a limitation vector of actuators. The energy function *E*(*k*) is defined as

(14) $$E(k)=\frac{1}{2}e{\left(k\right)}^{2}$$

and the parameters of PID are updated based on the following rule:

(15) $$K_{c}(k)=\{\begin{array}{ll} K_{c}(k-1), & f\left\|\frac{\partial y}{\partial u}\right\|\geq \gamma \\ K_{c}(k-1)+\Delta K_{c}(k), & \text{otherwise} \end{array}$$

where *∂y/∂u* is the Jacobian information of the controlled process, which can be identified by adopting a neural network model. *γ* is a threshold, by which the tuning Equation (15) can prevent PID gain parameters from being increased rapidly due to the effect of external strong interference. *δK_c_*(*k*) is the increment of the gain vector *K_c_*(*k*), adjusted based on the negative gradient method as follows:

(16) $$\Delta K_{c}=-\eta \frac{\partial E}{{\partial K}_{c}}=-\eta \frac{\partial E}{\partial y}\frac{\partial y}{\partial u}\frac{\partial u}{\partial K_{c}}=\eta e(k)\frac{\partial y}{\partial u}e_{c}(k)$$

where *η* is a learning rate parameter matrix.

**Figure 4.**
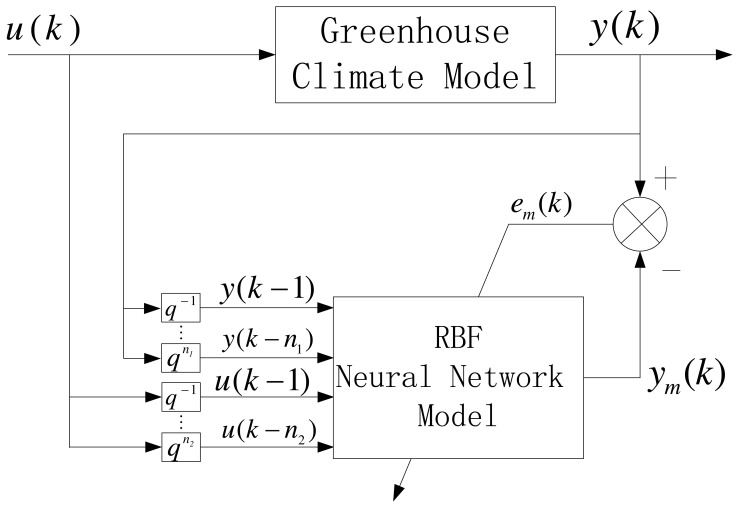
Process identification based on RBF network.

Because the RBF network has excellent adaptability, learning ability and powerful ability to approximate nonlinear functions, we adopt the identification structure as shown in Figure 4. The output vector of RBF neural network model is defined as:

(17) $$y_{m}(n)=\hat{f}(u_{1}(k-1),u_{2}(k-1),\ldots ,u_{1}(k-n_{1}),u_{2}(k-n_{1}),y_{1}(k-1),y_{2}(k-1),\ldots ,y_{1}(k-n_{2}))$$

where *f̂*(*·*) is an approximated nonlinear function of *f*(*·*), and *n_1_* and *n_2_* are the number of lag time steps. According to the universal approximation proposition [44], the *i^th^* RBF network node output *y_mi_*(*k*) will tend to the *i^th^* loop process output *y_i_*(*k*) when providing enough numbers of hidden layer nodes. Therefore, we can achieve the following formula by providing an appropriate hidden neuron:

(18) $$\frac{\partial y_{i}(k)}{\partial u_{i}(k)}\approx \frac{\partial y_{mi}(k)}{\partial u_{i}(k)}=\sum_{j=i}^{M}w_{j}h_{j}\frac{c_{ji}-I_{i}(k)}{\sigma_{j}^{2}}$$

where *i=* 1,2.

### 3.3. Jacobian Identification of RBF Network

The identification algorithm of Jacobian information of controlled process is stated below. The performance index function of controller is defined as:

(19) $$E_{m}(k)=\frac{1}{2}e_{m}{\left(k\right)}^{2}$$

where *e_m_*(*k*) is the error between the process actual output and RBF network model output, which is represented by

(20) $$e_{m}(k)=y(k)-y_{m}(k)$$

In order to minimize the error *e_m_*(*k*), a gradient descent method is adopted to adjust the weights, center vectors and center width parameters. The update equations for the RBF network parameters are given as:

(21) $$\Delta w_{j}(k)=-\frac{\partial E_{m}(k)}{\partial w_{j}(k)}=\frac{\partial E_{m}(k)}{\partial y_{m}(k)}\cdot \frac{\partial y_{m}(k)}{\partial w_{j}(k)}=e_{m}(k)h_{j}$$

(22) $$w_{j}(k)=w_{j}(k-1)+\eta_{w}(k)\Delta w_{j}(k)+\alpha_{w}(w_{j}(k-1)-w_{j}(k-2))+\beta_{w}(w_{j}(k-2)-w_{j}(k-3))$$

(23) $$\Delta \sigma_{j}(k)=e_{m}(k)w_{j}h_{j}\frac{{\left\|X-C_{j}\right\|}^{2}}{\sigma_{j}^{3}}$$

(24) $$\sigma_{j}(k)=\sigma_{j}(k-1)+\eta_{\sigma }(k)\Delta \sigma_{j}(k)+\alpha_{\sigma }(\sigma_{j}(k-1)-\sigma_{j}(k-2))+\beta_{\sigma }(\sigma_{j}(k-2)-\sigma_{j}(k-3)$$

(25) $$\Delta c_{ji}(k)=e_{m}(k)w_{j}\frac{x_{i}-c_{ji}}{\sigma_{j}^{2}}$$

(26) $$c_{ji}(k)=c_{ji}(k-1)+\eta_{c}(k)\Delta c_{ji}(k)+\alpha_{c}(c_{ji}(k-1)-c_{ji}(k-2))+\beta_{c}(c_{ji}(k-2)-c_{ji}(k-3))$$

where *α_w_, α_σ_, α_c_, β_w_, β_σ_* and *β_c_* are the corresponding momentum factors, and they can speed up convergence and prevent the network from falling into local minima. *η_w_*(*k*), *η_σ_*(*k*) and *η_c_*(*k*) are appropriate learning rate parameters. Unduely low learning rate makes the network learn very slowly, while excessively high learning rate makes the weights and objective function diverge. Therefore, we adopt the tuning method as follows:

$$\Delta e_{m}(k)=|e_{m}(k)|-|e_{m}(k-1)|$$

$$\eta_{w}(k)=\eta_{w}(k-1)-\xi \Delta e_{m}(k)$$

$$\eta_{\sigma }(k)=\eta_{\sigma }(k-1)-\xi \Delta e_{m}(k)$$

$$\eta_{c}(k)=\eta_{c}(k-1)-\xi \Delta e_{m}(k)$$

where *ξ* is a constant coefficient and we select here *ξ* as 0.0001. When the error signal *e_m_* increases with iteration process, the learning rate parameters are decreased, and conversely, the learning rate parameters are increased when the error signal *e_m_* decreases.

## 4. Simulations and Results

In order to verify the efficiency and performance of the proposed Neuro-PID control scheme, a series of simulations are presented in the present section. For this example, we consider a greenhouse of surface area 1,000 *m*^2^ and a height of 4 *m*. The greenhouse has a shading screen that reduces the incident solar radiation energy by 60%. The maximum water capacity of the fog system is 26 *g*[*H_2_O*]*min-^1^m^−3^*. Maximum ventilation rate corresponds to 20 air changes per hour (22.2 *m*^3^*s*^−1^). The parameters of the greenhouse climate model are shown in Table 1. Moreover, the initial values of indoor air temperature and humidity ratio are 25 °C and 20 g[*H*_2_*O*]/kg[air], respectively. Here, the threshold *γ* = 0.0085, the lag number *n_1_ = n_2_ =* 2, and the sampling time is 1 min.

**Figure 5.**
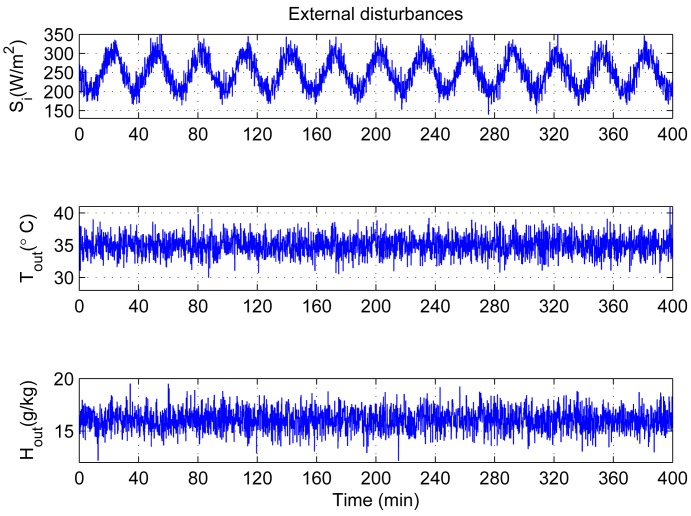
Changes of outdoor climate.

Considering the external climatic fluctuation within a small range during short time, solar radiation *S_i_*, outdoor temperature *T_out_* and humidity ratio *H_out_* change in random ways as shown in Figure 5 to represent external disturbances. Another important consideration is the many learning rates and momentum factors, which determine how much we change the corresponding modifier formulas at each step. For convenience, we select here the fixed parameters for each control loop as follows:

$$\begin{array}{l} \eta =\left(\begin{array}{ccc} 1.5 & 0 & 0\\ 0 & 0.5 & 0\\ 0 & 0 & 1.2 \end{array}\right)\\ \alpha_{w}=\alpha_{\sigma }=\alpha_{c}=0.1\\ \beta_{w}=\beta_{\sigma }=\beta_{c}=0.15 \end{array}$$

and the initial learning rate parameters

$$\eta_{w}=\eta_{\sigma }=\eta_{c}=0.05$$

**Figure 6.**
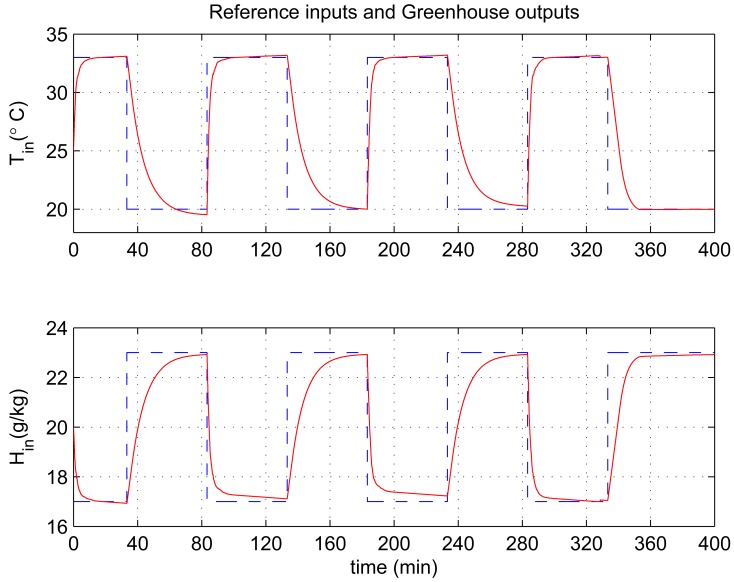
Neuro-PID controller tracking trajectory of square-wave for temperature and humidity ratio.

**Figure 7.**
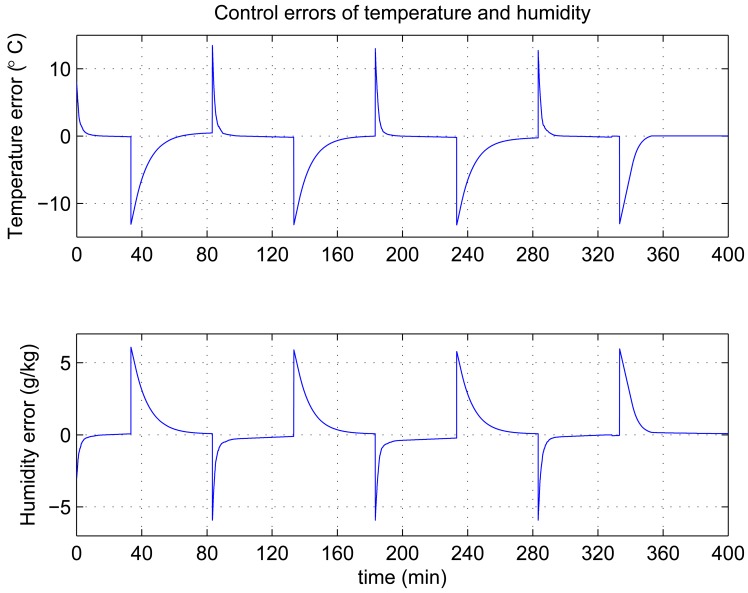
Variation of Neuro-PID control errors during the tuning process.

**Figure 8.**
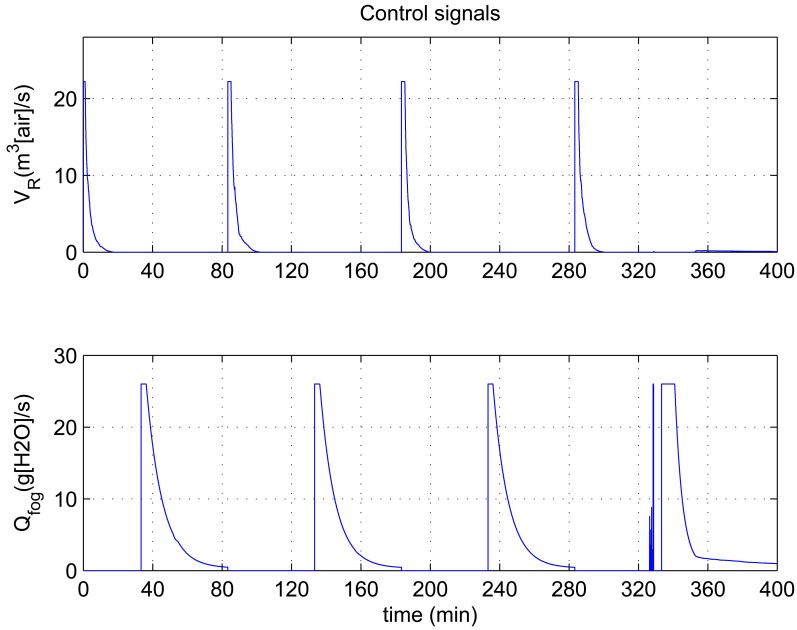
Variation of Neuro-PID control signals during the tuning process.

**Figure 9.**
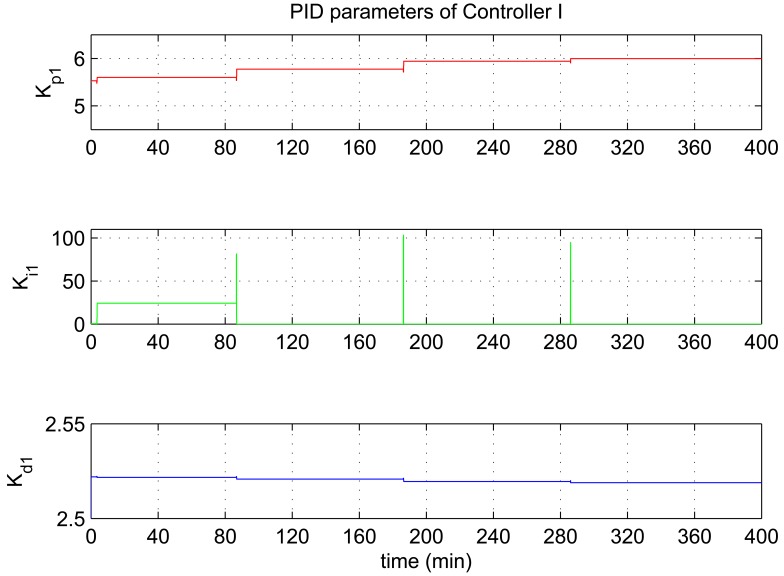
Variation of the PID parameters for Controller I during the tuning process.

**Figure 10.**
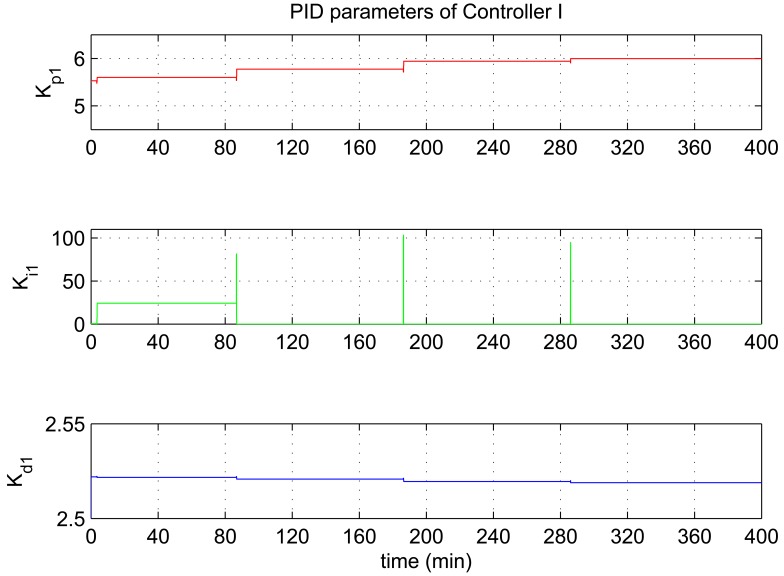
Variation of the PID parameters for Controller II during the tuning process.

**Figure 11.**
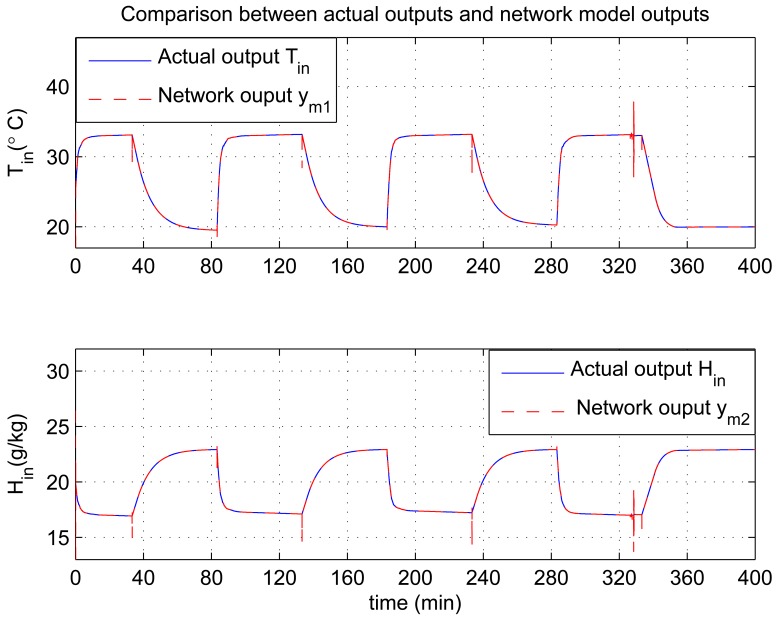
Temperature and humidity matching conditions between the actual output and RBF network model output.

In this experiment, the ability of the system to perform adaptive control and smooth closed-loop response to set-point changes is demonstrated. We consider a pair of square-wave reference inputs to test its control performance. Indoor air temperature set-point changes between 33 and 20 °C, while the humidity ratio set-point changes between 17 and 23 g [*H*_2_*O*]/kg[air] (which corresponds to a relative humidity change between 60% and 76%). The corresponding responses for set-point square-wave changes in temperature and humidity ratio are shown in Figure 6, and the corresponding errors and control signals are illustrated in Figures 7 and 8. The results from the figures show that the proposed adaptive control scheme performs well without strong overshoot and the controlled process is smooth. Figures 9 and 10 show that the gain parameters of the Neuro-PID controller vary with the set-point changes during the tuning process. The PID parameters can be adjusted automatically to adapt to outside environmental or climatic changes. The matching condition of the system actual output and RBF network output are illustrated in Figure 11. The results show that the outputs of neural network model are approximate to the controlled process outputs, which means that the RBF network outputs converge to the process outputs in the proposed method. On the whole, system outputs track the reference inputs well, and the Neuro-PID controller demonstrates good adaptive and tracking performance.

To demonstrate the superiority of the proposed control scheme, we compare the results with an offline tuning method that uses GA to search the PID gain parameters according to the performance requirements. Each chromosome consists of six separate strings (*i.e.*, *K_p_*_1_, *K_i_*_1_, *K_d_*_1_, *K_p_*_2_, *K_i_*_2_, *K_d_*_2_). The objective function considered is based on the error criteria, and we take ITAE as a measure of performance index. Considering the two loop control system of greenhouse climate, the performance index (*i.e.*, fitness function) is formulated as follows:

(27) $$J=\sum_{k=1}^{N}(t(k)\sum_{i=1}^{2}\left|e_{i}(k)|\right)$$

where *e_i_*(*k*) is the error signal of the *i^th^* loop, which is provided by Equation (10). *t*(*k*) represents the time of the *k^th^* iterative, *N* is the total number of iteration.

Therefore, the optimization problem can be formulated as follows:

Find *ind* = [*K_p_*_1_,*K_i_*_1_,*K_d_*_1_,*K_p_*_2_,*K_i_*_2_,*K_d_*_2_]
to optimize *J*(*ind*)
subject to 1 *≤ ind*(*i*) *≤* 100
for *i*= 1,*…*,6.

The parameters of GA optimization are shown in Table 2. The best PID gain parameters achieved by GA optimization are *K_p_*_1_ = 21.7780,*K_i_*_1_ = 40.1346,*K_d_*_1_ = 86.7532, *K_p_*_2_ = 98.4024,*K_i_*_2_ = 47.4968 and *K_d_*_2_ = 97.0353, respectively.

**Figure 12.**
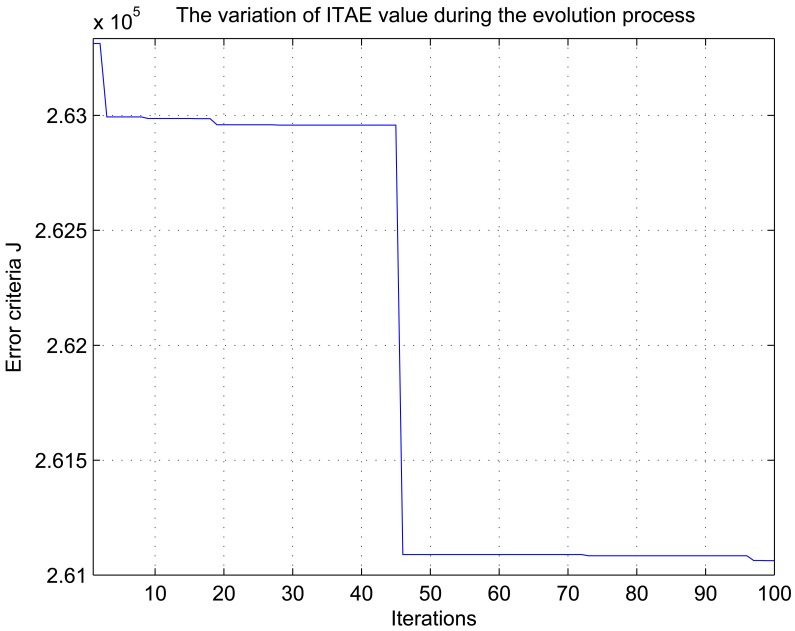
Variation of the objective function value during the evolution process for GA optimization.

**Figure 13.**
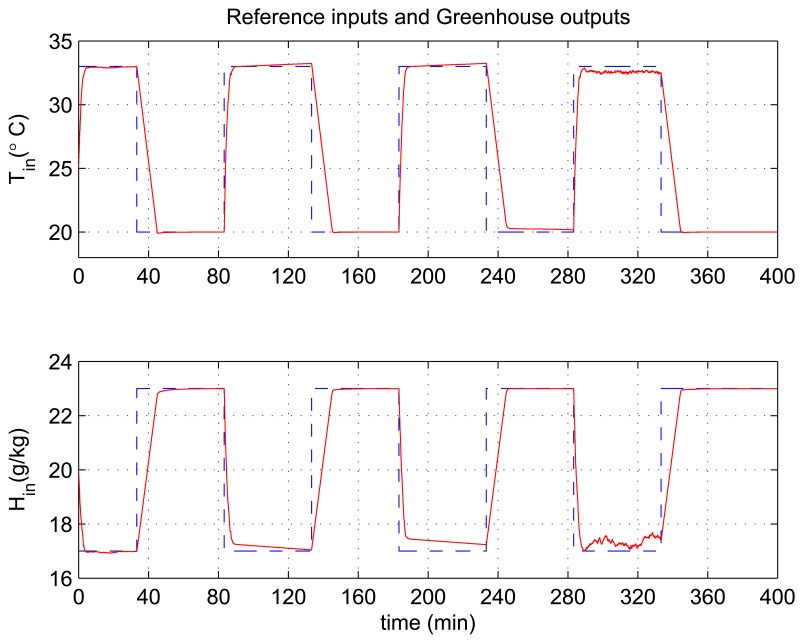
Conventional PID controller tracking trajectory of square-wave for temperature and humidity ratio, *K_p_*_1_ = 21.7780,*K_i_*_1_ = 40.1346,*K_d_*_1_ = 86.7532, *K_p_*_2_ = 98.4024, *K_i_*_2_ = 47.4968,*K_d_*_2_ = 97.0353.

**Figure 14.**
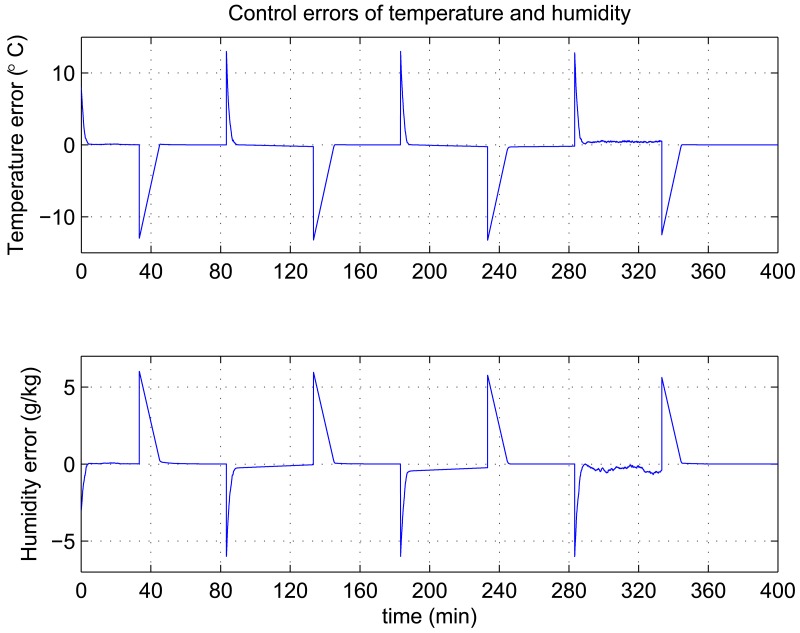
Variation of conventional PID control errors during the tuning process.

**Figure 15.**
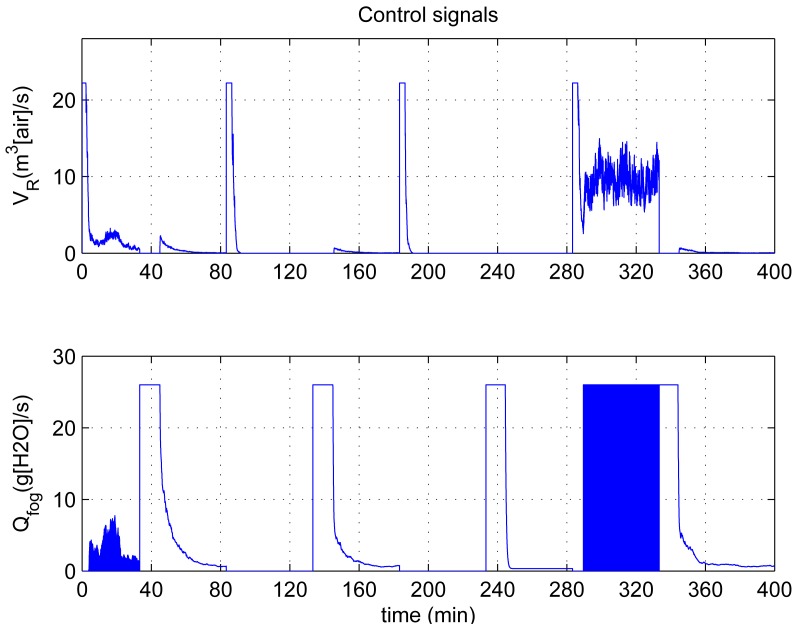
Variation of conventional PID control signals during the tuning process.

Figures 12–15 show the variation of ITAE value, the tracking trajectory of square-wave, the variation of control errors and the corresponding control signals for the conventional PID controller during the offline tuning process, respectively. To discriminate the performance of the two methods, the tracking error are given in Table 3. Results show that the offline tuning scheme can also achieve the tracking performance, and the mean error and standard deviation are even smaller than those of the online scheme. However, the tracking trajectory is not smooth and the control process generates oscillating control signals at about 290–315 min by the use of an offline tuning scheme. Its control performance is less smooth compared with the proposed Neuro-PID controller. The actual cause can be interpreted as follows: the offline tuning method cannot adapt to the external climatic fluctuation. Changes of solar radiation *S_i_*, outdoor temperature *T_out_* and humidity ratio *H_out_* caused control performance degradation. Besides, the offline tuning scheme is time-consuming and heavily dependent on the computation time of the GA optimization, and it can not be applied to the real-time control.

From the above results, it can be seen that the proposed adaptive Neuro-PID control scheme has good control effect such as good adaptability, strong robustness while achieving satisfactory control performance for the complex and nonlinear greenhouse climate control system. Compared with the offline tuning method that uses GA optimization, the proposed adaptive strategy has the following advantages. Firstly, it has a better set-point tracking performance. Secondly, it has smoother control process characterized by smaller oscillatory amplitudes. Thirdly, it can be applied to online real-time control for the complex greenhouse climate system. Lastly, and most important of all, it adapts well to external climatic fluctuation.

## 5. Summary and Conclusions

In this paper, we combined RBF network with a conventional PID controller to develop a hybrid control strategy for greenhouse operation. A model for nonlinear conservation laws of enthalpy and matter between numerous system variables affecting the greenhouse climate was formulated, and the corresponding control model was presented and discussed. Considering the characteristic of nonlinear learning ability of the RBF network, RBF network was used to tune and identify all PID gain parameters online and adaptively. The adaptive tuning method based on Jacobian identification of RBF network was derived, and the implementation of the Neuro-PID controllers is presented. The proposed Neuro-PID control scheme was validated for the complex greenhouse climate control in terms of set-point tracking and disturbance rejection by a simulation experiment. Adaptability, tracking and smooth closed-loop response to set-point changes was demonstrated by tracking square wave trajectory in this experiment. We compared the proposed adaptive online tuning method with the offline tuning scheme that employs GA to search the optimal gain parameters based on the error criteria. Results show that the proposed adaptive control strategy has better adaptability, robustness, and more satisfactory real-time control performance compared with the offline tuning scheme using GA optimization.

The results suggest that the proposed adaptive hybrid control scheme by combining RBF network with a conventional PID controller is a promising method with the following features. Firstly, it has a satisfactory control performance characterized by adaptability, robustness and good set-point tracking. Secondly, it can achieve the real-time online control of various MIMO systems. Thirdly, it can be applied in nonlinear dynamic control systems such as greenhouse climate system. This new system may provide a valuable reference to formulate environmental control strategies for actual application in greenhouse production. The approach is not limited to greenhouse applications and could easily be extended to other applications. Therefore, further improvement and implementation of the system will be achieved in the near future.

## Figures and Tables

**Table 1.** Greenhouse model parameters.

**Parameters name**	**unit expression**	**values**
***U****A*	*kWK*^−^*^1^*	25
α		0.129524267
*β_T_*	*kgmin^−1^m^−2^*	0.015
*V_T_*	*m^3^*	0.65*V*
*V_H_*	*m^3^*	0.65*V*
*λ*	*Jg^−1^*	2,257
*ρ*	*kg*[air]*m*^−3^	1.2
*C_p_*	*Jkg^−1^K^−1^*	1,006

**Table 2.** Parameters of GA optimization.

**Description**	**values *or* types**
Population size	60
Number of generations	50
Bit length of the considered chromosome	20
Crossover type	Single point crossover
Crossover probability	0.6
Mutation type	Uniform mutation
Mutation probability	0.001

**Table 3.** Performance Comparison between RBF online tuning and GA offline optimization.

**Methods**	**Temperature error (°C)**		**Humidity error Mean (*g*[*H*_2_*O*]*min^-^*^1^*m^-^*^3^)**	
	
	**Mean**	**STD**	**Mean**	**STD**
RBF online tuning	−0.9123	2.8812	0.3660	1.3550
GA offline optimization	−0.5870	2.7252	0.1765	1.2785
